# Interactive effects of spectral quality and trace metal availability on the growth of *Trichodesmium* and *Symbiodinium*

**DOI:** 10.1371/journal.pone.0188777

**Published:** 2017-11-30

**Authors:** Irene B. Rodriguez, Tung-Yuan Ho

**Affiliations:** 1 Research Center for Environmental Changes, Academia Sinica, Taipei, Taiwan; 2 Institute of Oceanography, National Taiwan University, Taipei, Taiwan; University of Connecticut, UNITED STATES

## Abstract

Light and trace metals are critical growth factors for algae but how the interdependence of light quality and metal availability affects algal growth remains largely unknown. Our previous studies have demonstrated the importance of Ni and Fe on the growth of *Trichodesmium* and *Symbiodinium*, respectively, two important marine primary producers inhabiting environments with high light intensities. Here, we investigated the effects of light quality and intensity with availability of either Ni or Fe on their growth. For *Trichodesmium*, we found that specific growth rates for high Ni treatments were all significantly higher than in corresponding low Ni treatments with varying light quality and intensity. The inhibitory effect of low intensity red light was also countered by sufficient Ni supply. For *Symbiodinium*, we found that growth rates and biomass were reduced by 75% under low intensity red light and the stress can only be partially relieved by sufficient Fe supply. The results show that trace metal availability plays an important role in relieving the stress induced by low red light condition for both *Trichodesmium* and *Symbiodinium* although the cyanobacterium performs better in this growth condition. The difference may be attributed to the presence of phycocyanin, a unique pigment attuned to absorption of red light, in *Trichodesmium*. Our study shows that the concerted effects of light intensity and quality compounded with trace metal availability may influence the growth of photosynthetic organisms in the ocean.

## Introduction

Photosynthetic unicellular organisms are major primary producers in the ocean. Their biomass and community structure are controlled by various physical and chemical factors in the euphotic zone of the ocean. The euphotic zone, which is generally characterized by low nutrient availability, is restricted to the top 100 to 200 m where sunlight can penetrate [1]. In this zone, both the quantity and quality of solar radiation vary temporally due to diurnal or seasonal changes and spatially due to different depths and latitudinal positions that photosynthetic organisms occupy. Thus, photosynthetic organisms have evolved to possess unique capacities to survive in habitats with dramatically different light intensity, spectral quality, and nutrient supply. *Trichodesmium* and *Symbiodinium* are two different marine primary producers that survive in environments with extremely variable light conditions. *Trichodesmium* is a major diazotrophic cyanobacterium in open oceans while *Symbiodinium* comprises the largest group of symbiotic dinoflagellates in coral reef ecosystems. Although they are phylogenetically distinct, their habitats are both characterized by strong light intensities, which may cause photoinhibition of the photosynthetic system. *Trichodesmium* is considered a major contributor of new production in oligotrophic tropical and subtropical oceans where light intensity in the surface water can reach 2000 μmol photons m^-2^ s^-1^ during midday [2,3]. As for *Symbiodinium*, the quality and intensity of light it experiences is largely controlled by its coral host [4,5]. Even though the dinoflagellate resides in the endoderm of its host, it is still exposed to intense light that is susceptible to extreme light intensity fluctuations due to tidal forcing and other physical factors [6].

To carry out photosynthesis, organisms use assorted strategies including utilization of various pigments to acquire maximum energy from available light [7]. The energy is then passed to photosystem reaction centers through electron transfer chains to produce photosynthates while unavoidably generating reactive oxygen species (ROS) as by-products [8]. Cellular regulation of ROS stress is thus essential during photosynthesis to maintain high microalgal growth [8]. In addition to common chlorophyll pigments, *Trichodesmium* also uses phycocyanin and phycoerythrin as its photosynthetic pigments while *Symbiodinium* has a distinct peridinin-chlorophyll *a*-protein for its photosynthetic requirements [9–11]. The presence of these specific photosynthetic pigments in organisms compensates for the inability of chlorophyll pigments, which mainly cover a wide region at the range for blue light and a relatively narrow peak between 680 and 700 nm, to absorb other wavelengths of light. Previous laboratory culture studies showed that *Trichodesmium* growth and nitrogen fixation rates were much lower in red light than in blue light [12,13]. For *Symbiodinium*, several laboratory culture studies showed that dinoflagellates flourished best in blue light while growth was stunted in red light [14,15]. In early studies conducted by Kinzie et al. (1984), they observed that *in vitro* cultures of *Symbiodinium microadriaticum* isolated from *Montipora verrucosa* behaved similarly to intact coral-algal symbionts exhibiting better growth in blue light compared to that in green or red light [14]. These previous studies demonstrate that light quality is a significant factor determining the growth and sustainability of various microalgal populations.

In our prior work, we have demonstrated that Ni availability may interact with light intensity to affect growth and nitrogen fixation of *Trichodesmium* [16–18]. *Trichodesmium* can simultaneously carry out photosynthesis and nitrogen fixation during daytime despite incompatibility of the two processes owing to sensitivity of nitrogenase to oxygen and other ROS generated as by-products of photosynthesis [19]. We have posited that nickel-containing superoxide dismutase (Ni-SOD) is essential for *Trichodesmium* to regulate cellular ROS levels to protect nitrogenase and enable it to concomitantly fix carbon and nitrogen. Similar to *Trichodesmium*, *Symbiodinium* is also subjected to elevated ROS levels. The production and accumulation of ROS is widely regarded as a major cause of coral bleaching, which is associated with the loss of endosymbionts or loss of their photosynthetic pigments [20–22]. Interestingly, a genomic study revealed that the dinoflagellate *Symbiodinium kawagutii* contains the genes required for synthesis of four different SODs with Fe, Mn, Cu/Zn, and Ni as cofactors [23]. We found in our recent study that *Symbiodinium* requires high concentrations of Fe to maintain maximum growth rates under relatively high light conditions [24]. The high Fe requirement of *Symbiodinium* may be attributed to Fe necessitated in photosynthetic systems and anti-oxidative enzymes including Fe-SOD, catalase, and peroxidases. Within the coral holobiont, this Fe requirement may be compounded by the need to adjust to hyperoxic conditions in the coral endoderm, which is countered by both host and endosymbiont via production of their respective anti-oxidative enzymes [6]. The intense ROS stress in *Symbiodinium* indicates that Fe may be a limiting factor in coral reef ecosystems. Our prior results combined with that of others [12–24] point to the possibility of interactive effects between physical and chemical growth factors to influence growth of organisms like *Trichodesmium* and *Symbiodinium*.

Owing to their different pigment composition but comparable high light habitats, *Trichodesmium* and *Symbiodinium* were chosen for this study. Since both specific light quality and increasing light intensity may elevate ROS production, both of these conditions were varied in this study. We propose that higher Ni or Fe availability may counteract the inhibitory effect of high intensity red or blue light on the growth of *Trichodesmium* and *Symbiodinium*, respectively. To interrogate the hypotheses, we carried out laboratory cultures of both organisms in two different intensities of red and blue light, and compared their growth performance when subjected to full spectrum white light. To test the effect of metal availability, we carried out two different sets of Ni and Fe treatments for *Trichodesmium* and *Symbiodinium*, respectively. The results from this work will bridge the knowledge gap in how light quality and intensity interact with metal availability to influence growth of two important microalgae.

## Materials and methods

### *Trichodesmium erythraeum* treatments

Nonaxenic cultures of *Trichodesmium erythraeum* IMS101 (*Trichodesmium* from here onwards), obtained from National Center for Marine Algae and Microbiota, were kept in 1 L acid washed polycarbonate bottles with trace metal-defined culture medium containing 20 μM ethylenediaminetetraacetic acid (EDTA) [16]. The total dissolved concentration in high and low Ni treatments were 10 and 100 nM resulting in expected inorganic concentrations (Ni′) of 6.7 pM and 67 pM in culture media, respectively (calculated using MineQL version 4.0) [25]. These Ni treatments are designated as low Ni or high Ni treatment from here onwards. Other trace metals were added at dissolved total concentrations of 10 nM for Mn, Zn, Co, and Cu, 100 nM for Mo, and 400 nM for Fe. For different light treatments, we used either 250 or 680 μmol photons m^-2^ s^-1^ of red, blue or full spectrum white light referred to as low or high red, blue or white light treatment from here onwards. These different light conditions and Ni concentrations resulted in a total number of 12 different treatments.

### *Symbiodinium kawagutii* treatments

Non-axenic cultures of *Symbiodinium kawagutii* strain CCMP2468 (*Symbiodinium* from here onwards) were grown in 500 mL acid-washed polycarbonate bottles with trace-metal defined medium modified from the original L1 medium recipe [24]. This strain belongs to phylogenetic clade F1 and was isolated from the scleractinian coral *Montipora verrucosa* in the North Pacific Ocean (21.25°N 158°W, Hawaii, USA). Seawater collected from surface water of the South China Sea South East Asia Time-Series (SEATS, 18°N 116°E) station was used for medium preparation. We carried out two sets of treatments with differing Fe concentrations equivalent to total concentrations of 50 or 250 nM, which resulted in expected inorganic Fe concentrations (Fe′) of 250 or 1250 pM with the addition of 20 μM EDTA (calculated using MineQL version 4.0) [25]. The Fe treatments are referred to as low Fe or high Fe treatment from here onwards. Other trace metals were supplied as follows: 10 nM Cu and 100 nM of Mn, Zn, and Ni. For light treatments, we also used either 250 or 680 μmol photons m^-2^ s^-1^ of blue, red or full spectrum white light. These different light conditions and Fe concentrations led to a total of 12 different treatments.

### Acclimatization procedure

Acclimatization of *Trichodesmium* or *Symbiodinium* to the desired growth conditions were achieved as follows: stock cultures (2 mL of *Trichodesmium* and 1 mL of *Symbiodinium*) were inoculated in PC culture bottles (1L for *Trichodesmium* and 500 mL for *Symbiodinium*) with fresh medium containing low or high metal availability (6.7 or 67 pM Ni′ for *Trichodesmium* and 250 or 1250 pM Fe′ for *Symbiodinium*). Once the cell density reached maximum biomass, the same volume of inoculum was transferred to fresh medium with similar Ni or Fe availability. Two sets of bottles for each metal condition were prepared to acclimate one set each in low light or high light intensity (250 or 680 μmol photons m^-2^ s^-1^). The cultures were allowed to reach maximum biomass before transferring the same volume of inoculum to further three different light quality conditions of red, blue or full spectrum white light, which makes the total treatments to 12 different growth conditions. For *Trichodesmium*, the cells were transferred again to fresh medium and subjected to similar conditions for two more passages before carrying out the final experiment wherein triplicates were inoculated from the culture bottle subjected to similar growth conditions. For *Symbiodinium*, only one passage was carried out before performing the final experiment because previous work in the laboratory showed that one passage was enough for the dinoflagellate to show the physiological effects.

### Other culture conditions

Both artificial and natural seawater were filtered using Whatman^®^ Polycap filters, passed through a column packed with Chelex-100^®^ resin to remove trace metal contents, and filter-sterilized using 0.22 μm pore size filters prior to use. In both algal cultures, the total initial concentration of phosphate was 50 μM. The vitamin B mixture, composed of thiamine, biotin and cyanocobalamin, was added to have final concentrations in the medium equivalent to 300, 2.0, and 4.0 nM, respectively. The cultures with *Trichodesmium* were ensured to be free of fixed nitrogen while those with *Symbiodinium* contained 800 μM of nitrate. Stock solutions of phosphate, nitrate, thiamine, and biotin were all passed through columns packed with Chelex-100^®^ to remove metal contaminants. The stock cyanocobalamin solution was not passed through the column to avoid loss of cyanocobalamin. All stock solutions were filter-sterilized using 0.22 μm pore size filters prior to use.

Bottles and plastic materials used for culturing and other related work were carefully washed with 2% Micro-90^®^ solution, rinsed, soaked with 10% hydrochloric acid solution, and rinsed thoroughly with ultrapure water prepared using a Milli-Q system. All necessary procedures were performed in a class 100 trace-metal clean laboratory. All treatments were carried out in triplicates, which were inoculated from a source that was acclimatized to respective growth conditions. The inoculation volume used was set at 1/500 to ensure minimum carry-over effects of trace metals. Cultures were kept in a temperature-controlled growth chamber set at 26°C. The light and dark periods were programmed under a square-wave 12:12 h light:dark regime with different light spectral treatments attained using red (China Electric, wavelength covering 630 to 670 nm and a peak at 655 nm), blue (China Electric, wavelength covering 420–480 nm and a peak at 430 nm), and full-spectrum white light fluorescent lamps (Philips). Different light intensities were achieved by placing culture bottles at appropriate distances from the light source and intensity was validated by measuring photon flux density using a submersible radiometer (Biospherical Instruments Inc. QSL 2100). Other culture information are described in detail in previous studies [16,24].

### Growth rates and intracellular metal quotas

Algal growth was monitored using a Beckman Coulter Counter Multisizer 3 with a 100 μm aperture tube for a period of 18–20 days, in which several treatments have attained stationary growth. Biomass of *Trichodesmium* and *Symbiodinium* were monitored by measuring total cellular volume per mL and cell number per mL, respectively [16]. The use of Coulter Counter for monitoring changes in cellular volume of *Trichodesmium* has been explained in detail in a previous report [16]. Other more robust methods are available for growth monitoring of filamentous phytoplankton. However, we find that the use of Coulter counter is a reliable method that offers a precision within 5% and is suitable for cellular volumes down to 5 × 10^3^ μm^3^ mL^-1^, which corresponds to presence of 1–2 trichomes per mL of culture by assuming 10–20 cells in one trichome.

Growth rates and intracellular metal quotas were determined while cells were in exponential stage of the growth period, between days 5 to 13 for *Trichodesmium* and 5 to 12 for *Symbiodinium*. Intracellular metal quotas were determined using cells harvested by filtration onto acid-washed polycarbonate filters (25 mm diameter, 5 and 2 μm pore size for *Trichodesmium* and *Symbiodinium*, respectively) during light phase of the photoperiod. Filtered cells were washed with ultrapure water and acid-digested prior to elemental analysis using HR-ICPMS (Element XR, Thermo Scientific). Intracellular Fe quotas for *Symbiodinium* were evaluated when biomass in individual treatments was about 2 × 10^5^ cells mL^-1^. Metal quotas were normalized against phosphorus as the biomass indicator and metal uptake rates were evaluated by multiplying intracellular quotas with corresponding growth rates in respective treatments. Procedural filter blanks were subjected to the same digestion, dilution, and analysis. The blank values were subtracted from sample measurements. Metal net use efficiency (NUE) was calculated by dividing specific growth rates with corresponding metal (M) to P ratios resulting in a value with unit of mol P mmol^-1^ M day^-1^ [26].

### Nitrogen fixation by *Trichodesmium*

The amount of nitrogen fixed by cultures supplied with 67 pM Ni′ was estimated using the acetylene reduction method [27] following steps outlined elsewhere [16]. All acetylene reduction assays were carried out while cells were in exponential phase of growth and when biomass was higher than 1 × 10^6^ μm^3^ mL^-1^ because previous experiments in our laboratory showed that this represents the minimum biomass that may be subjected to the assay. The cultures supplied with 6.7 pM Ni′ were not subjected to the assay because the cellular volumes in several treatments were lower than 1 × 10^6^ μm^3^ mL^-1^. Briefly, 10 mL aliquots of cultures at the exponential growth phase (duplicate samples were prepared from each of triplicate bottles per treatment for each time point) were transferred to 20 mL vials (Agilent). The vials were sealed using Teflon-coated caps and 2 mL air was drawn using a syringe, which was replaced by adding 2 mL of commercially available acetylene to initiate the experiment (99.9999% purity, Kouatsu Gas Kogyo, Japan). The vials were then incubated at the same growth conditions for 12 hours with time points set every two hours. After incubation for the desired period, 2 mL of headspace was drawn and the gaseous sample was subsequently analyzed for ethylene using an Agilent 7890A gas chromatograph equipped with a Poropak N column and a flame ionization detector. Estimation of N_2_ fixation was taken from acetylene reduction using a conversion ratio of 4:1 [27], and assumption that the Bunsen coefficient for ethylene is 0.084 [28]. Procedural blanks prepared by using vials filled with filtered YBC-II only were also subjected to the same acetylene reduction assay. The blank values were subtracted from sample measurements. The amount of nitrogen fixed is presented with the unit pmol N_2_ cell^-1^ by taking into account the total cellular volume in 10 mL aliquots subjected to the assay. The number of cells present in 10 mL aliquots were determined using the conversion factor of 250 μm^3^ per cell obtained in prior work [16]. Additionally, for *Trichodesmium*, NUE with respect to N_2_ fixed (Ni-NUE-N_2_) was calculated by dividing nitrogen fixation rates (pmol N_2_ cell^-1^ hr^-1^) with metal to cell ratios (pmol Ni cell^-1^) [26].

### Statistical analyses

One-way analysis of variance (ANOVA) was conducted to determine whether the results obtained in culture experiments on the effects of light quality, light intensity, and metal availability were significantly different or not (LSD, 0.05; SPSS).

## Results

### Effect of Ni′ and light conditions on *Trichodesmium* growth

**Fig 1:**
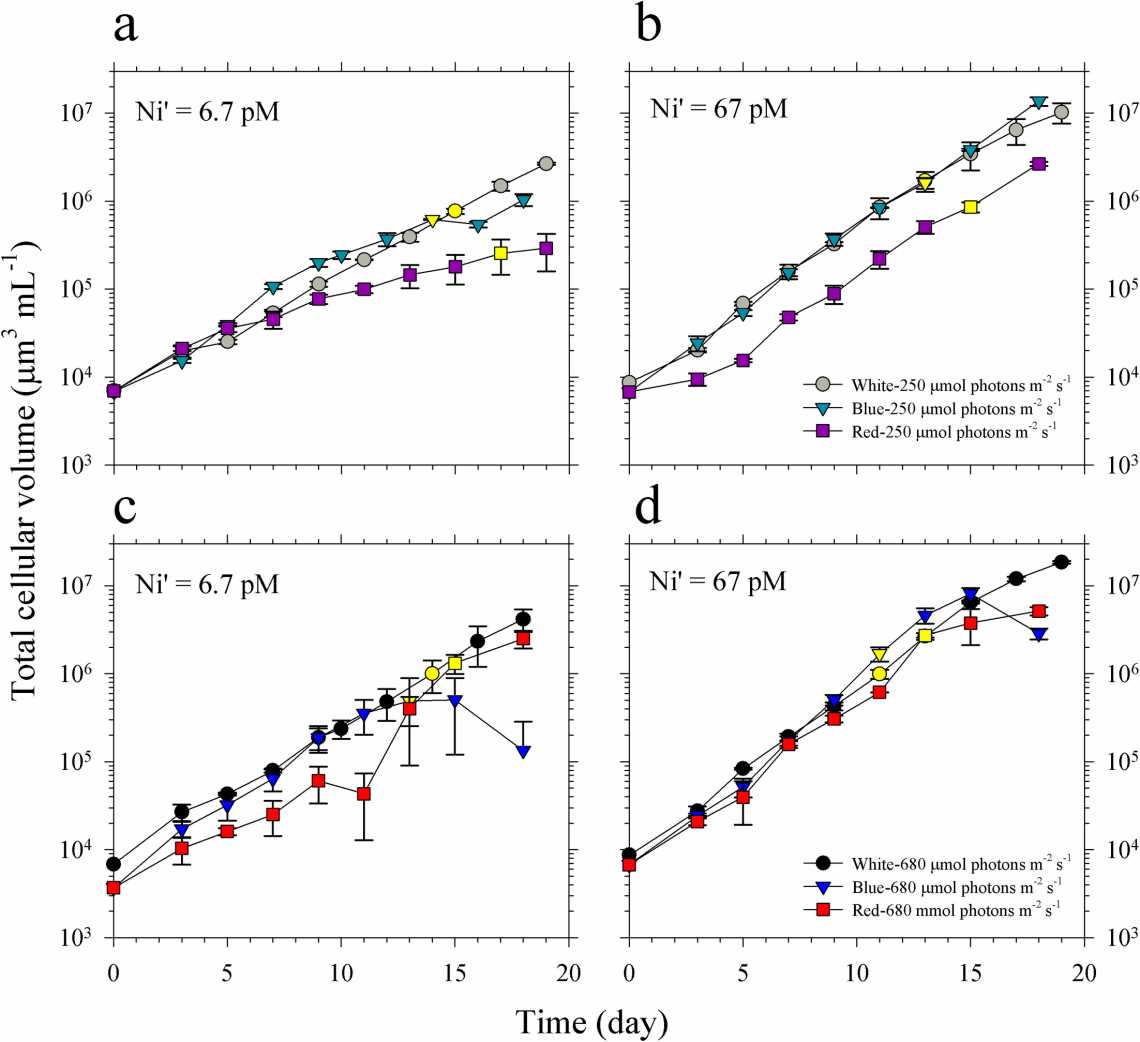
Growth curves and cellular volumes of *Trichodesmium* cultures grown in blue, red and full spectrum white light. **(a-b)** Growth curves of cultures grown in light supplied at 250 μmol photons m^-2^ s^-1^, and 6.7 and 67 pM Ni′, respectively. **(c-d)** Growth curves of cultures grown in light supplied at 680 μmol photons m^-2^ s^-1^, and 6.7 and 67 pM Ni′, respectively. Yellow-filled symbols indicate when cells for intracellular metal quota analyses were harvested. Error bars represent standard deviation of the value obtained from triplicate culture bottles.

**Fig 2:**
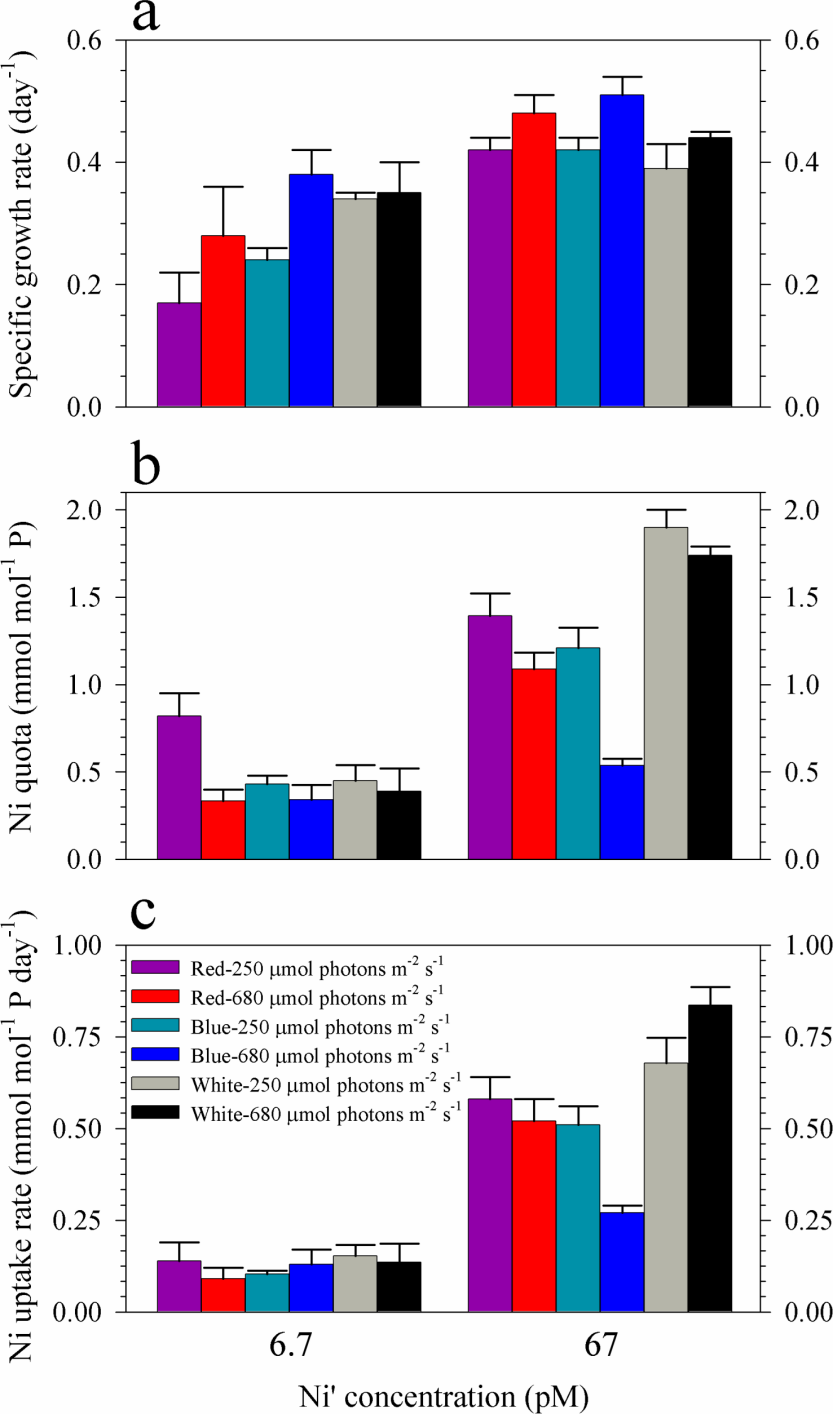
Specific growth rates, intracellular Ni quotas and uptake rates of *Trichodesmium* cultures grown in blue, red and full spectrum white light. **(a)** Specific growth rates, **(b)** Ni quotas, and **(c)** Ni uptake rates of *Trichodesmium* cultures grown in 6.7 or 67 pM Ni′ and subjected to light supplied at 250 or 680 μmol photons m^-2^ s^-1^. Error bars represent standard deviation of the value obtained from triplicate culture bottles.

The growth curves and rates of *Trichodesmium* showed that the three variables included in this study interact to influence the growth of *Trichodesmium* (Figs 1 and 2A, and Table 1). The results show that Ni availability affects phytoplankton growth, most notably in treatments subjected to low-red or low-blue light where slower growth was observed in cultures supplied with low Ni. In general, cultures supplied with 67 pM Ni′ exhibited higher growth rates compared with corresponding treatments containing 6.7 pM Ni′ and identical light quality and intensity (Fig 2A, Table 1). Considering the effect of light quality, blue light cultures of *Trichodesmium* demonstrated similar growth patterns and comparable biomass values, for most of the growth period, to white light cultures subjected to equivalent intensity and Ni concentration (Fig 1). For low intensity red light treatments, cultures showed distinct growth curves, with generally lower biomass values for most of the growth period, compared to white or blue light cultures in both sets of Ni treatments (Fig 1A and 1B). However, high red cultures presented similar growth patterns with other cultures (Fig 1C and 1D). Increase in light intensity resulted in improved growth especially in blue light cultures where the phytoplankton reached the stationary phase earlier when subjected to high intensity, which was also reflected in the calculated growth rates.

**Table 1:** Specific growth rates, intracellular Ni quotas, Ni uptake rates, and Ni net use efficiency of *Trichodesmium* cultures grown in blue, red and full spectrum white light. The values represent means ± SD (*N* = 3, *P* < 0.05, ANOVA, post hoc LSD).

Treatment Number	Treatment description	Ni availability (Ni′, pM)	Light intensity (μE m^-2^ s^-1^)	Light quality	Growth rate (day^-1^)	Ni quota (mmol Ni mol^-1^ P)	Ni uptake rate (mmol Ni mol^-1^ P day^-1^)	Net Use Efficiency (mol P mol^-1^ Ni day^-1^)	Net Use Efficiency (pmol N_2_ pmol^-1^ Ni hr^-1^)
1	low Ni-low red	6.7	250	red	0.17 ± 0.05^c^	0.82 ± 0.13^b^	0.14 ± 0.05^a^	0.21 ± 0.07^b^	-
2	low Ni-low blue	6.7	250	blue	0.24 ± 0.02^b^	0.43 ± 0.05^a^	0.10 ± 0.01^a^	0.56 ± 0.08^a^	-
3	low Ni-low white	6.7	250	white	0.34 ± 0.01^a^	0.45 ± 0.09^a^	0.15 ± 0.03^a^	0.76 ± 0.15^a^	-
4	low Ni-high red	6.7	680	red	0.28 ± 0.08^b^	0.33 ± 0.07^a^	0.092 ± 0.030^a^	0.84 ± 0.29^a^	-
5	low Ni-high blue	6.7	680	blue	0.38 ± 0.04^a^	0.34 ± 0.09^a^	0.13 ± 0.04^a^	1.1 ± 0.3^a^	-
6	low Ni-high white	6.7	680	white	0.35 ± 0.05^a^	0.39 ± 0.13^a^	0.14 ± 0.05^a^	0.90 ± 0.33^a^	-
7	high Ni-low red	67	250	red	0.42 ± 0.02^a^	1.4 ± 0.1^c^	0.58 ± 0.06^b^	0.30 ± 0.03^b^	89 ± 15^a^
8	high Ni-low blue	67	250	blue	0.42 ± 0.02^a^	1.2 ± 0.1^b^	0.51 ± 0.05^b^	0.35 ± 0.04^b^	247 ± 55^b^
9	high Ni-low white	67	250	white	0.39 ± 0.04^a^	1.9 ± 0.1^a^	0.68 ± 0.07^a^	0.21 ± 0.02^a^	121 ± 23^a^
10	high Ni-high red	67	680	red	0.48 ± 0.03^a^	1.1 ± 0.1^c^	0.52 ± 0.06^c^	0.44 ± 0.05^c^	252 ± 38^a^
11	high Ni-high blue	67	680	blue	0.51 ± 0.03^b^	0.54 ± 0.04^b^	0.27 ± 0.02^b^	0.95 ± 0.09^b^	92 ± 28^a^
12	high Ni-high white	67	680	white	0.44 ± 0.01^a^	1.7 ± 0.1^a^	0.84 ± 0.05^a^	0.25 ± 0.01^a^	168 ± 57^a^

Intracellular Ni quotas show that values obtained in treatments with 6.7 pM Ni′ were lower than in treatments with 67 pM Ni′ (Fig 2B, Table 1). It is worth noting that among low-Ni treatments, low-red cultures had a value that was almost twice as values determined in other cultures despite exhibiting the lowest growth rate. Also, among high-Ni treatments, high-blue cultures had a value that was two-fold to four-fold lower than in other treatments despite having the highest growth rate. The calculated uptake rates showed that values in treatments with low Ni were comparable regardless of light intensity or quality (Fig 2C, Table 1). Uptake rates for treatments with high Ni show that high-blue cultures had remarkably lower values compared to other treatments. The calculated Ni-NUE demonstrates that high blue light cultures had the highest values of 1.1 ± 0.3 and 0.95 ± 0.09 mol P pmol^-1^ Ni day^-1^ for low and high Ni treatments, respectively (Table 1).

### Effect of light quality and intensity on nitrogen fixation by *Trichodesmium*

**Fig 3:**
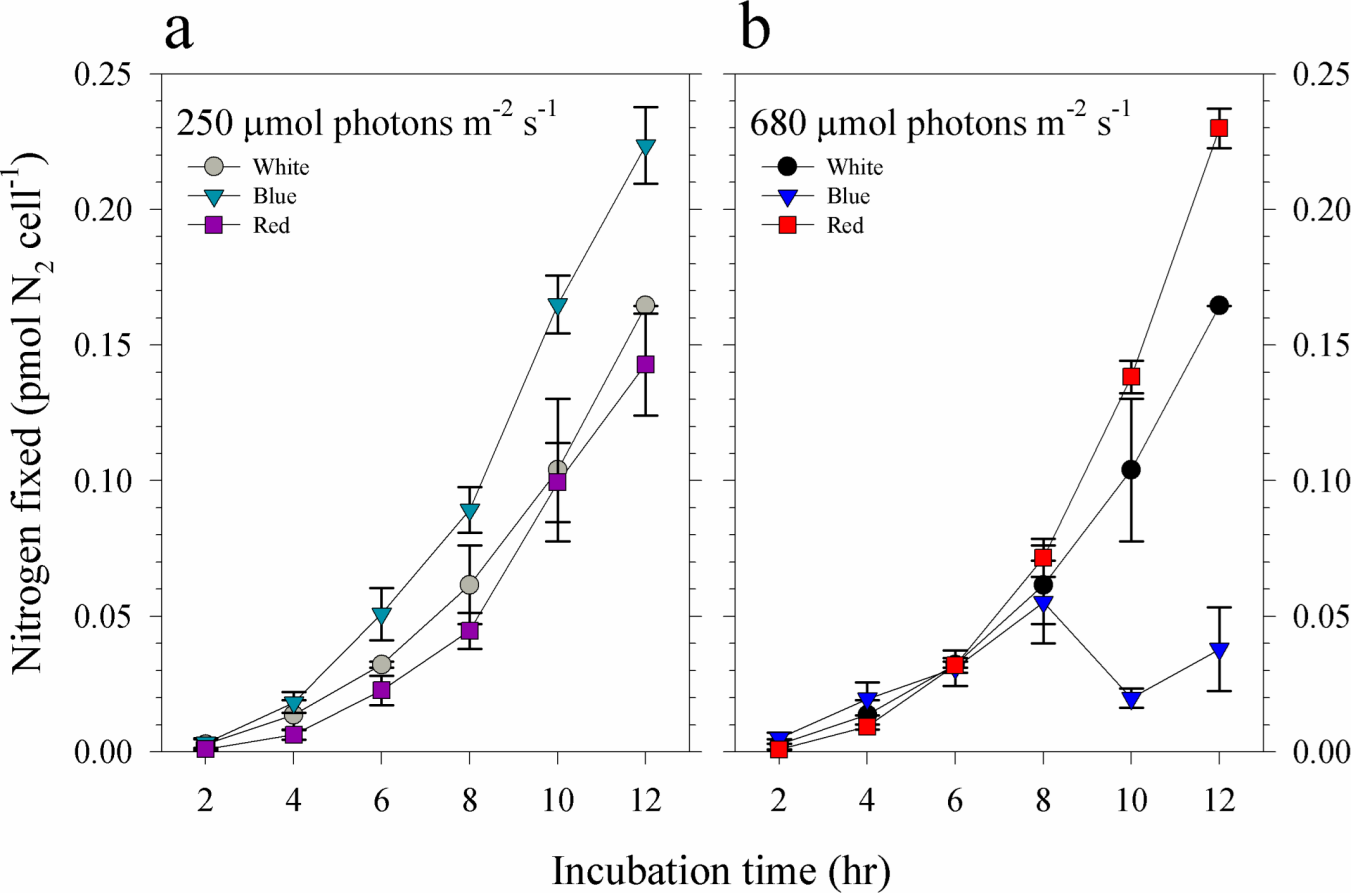
Nitrogen fixed by *Trichodesmium* cultures grown in 67 pM Ni′ and at different intensities of blue, red and full spectrum white light. Nitrogen fixed by cultures subjected to light supplied at **(a)** 250 μmol photons m^-2^ s^-1^ and **(b)** 680 μmol photons m^-2^ s^-1^. Levels of nitrogen fixed were estimated using the acetylene reduction assay. Error bars represent standard deviation of the value obtained from triplicate culture bottles.

Nitrogen fixation by *Trichodesmium* cultures supplied with 67 pM Ni′ was influenced by light intensity and spectral quality as determined by acetylene reduction assay (Fig 3). Results show that in low light intensity, blue light cultures fixed higher levels of N_2_ than both the red and white light cultures throughout the 12 h period, which coincided with day in the light:dark cycle (Fig 3A). These results differ from data observed in high light intensity where red light cultures fixed more nitrogen compared to blue and white light cultures (Fig 3B). Interestingly, at high light intensity, blue light cultures showed lower values for nitrogen fixed after 8 h of incubation. Comparison between cultures subjected to similar light quality but different light intensities resulted in different trends. Blue light cultures grown in low light intensity fixed more N_2_ while the opposite was observed for red light cultures. Comparable values were observed in white light cultures subjected to different light intensities. These trends were reflected in the calculated nitrogen fixation rates wherein blue light cultures had higher rates (0.030 ± 0.001 pmol N_2_ cell^-1^ hr^-1^) compared to red (0.021 ± 0.003 pmol N_2_ cell^-1^ hr^-1^) and white light cultures (0.022 ± 0.003 pmol N_2_ cell^-1^ hr^-1^) among low light treatments. In high light treatments, blue light cultures had lower rates (0.0066 ± 0.0016 pmol N_2_ cell^-1^ hr^-1^) compared to red (0.033 ± 0.001 pmol N_2_ cell^-1^ hr^-1^) and white light cultures (0.018 ± 0.002 pmol N_2_ cell^-1^ hr^-1^). The calculated Ni-NUE showed that high red cultures had the highest NUE with a value of 252 ± 38 pmol N_2_ pmol^-1^ Ni hr^-1^. A comparable value was observed in low blue cultures (247 ± 55 pmol N_2_ pmol^-1^ Ni hr^-1^) while the two lowest values were observed in low red and high blue cultures (Table 1).

### Effect of Fe, spectral quality, and intensity on *Symbiodinium* growth

**Fig 4:**
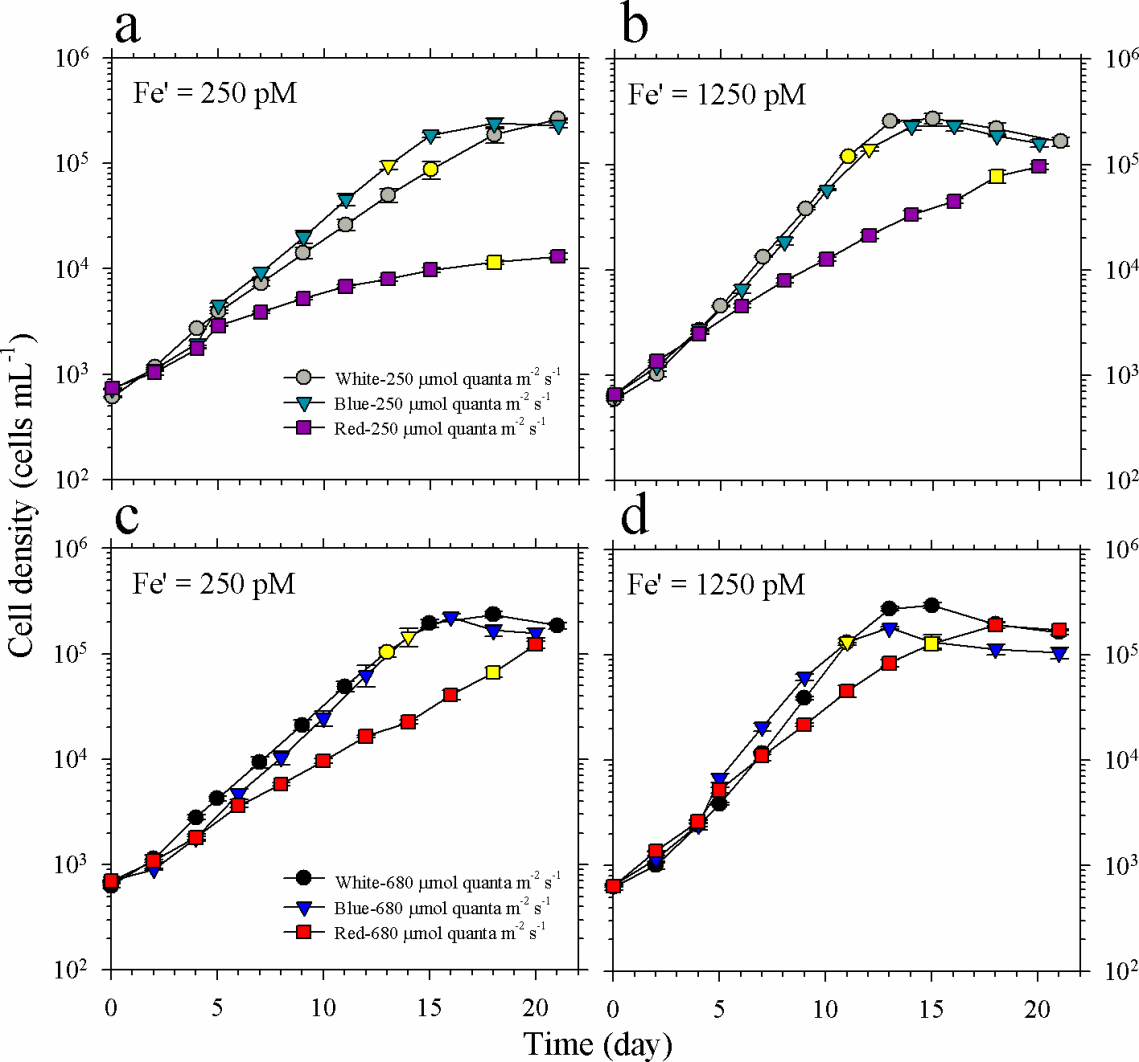
Growth curves of *Symbiodinium* cultures grown in different intensities of blue, red or white light and different Fe concentrations. **(a-b)** Growth curves of cultures grown in light supplied at 250 μmol photons m^-2^ s^-1^, and 250 and 1250 pM Fe′, respectively. **(c-d)** Growth curves of cultures grown in light supplied at 680 μmol photons m^-2^ s^-1^, and 250 and 1250 pM Fe′, respectively. Yellow-filled symbols indicate when cells for intracellular metal quota analyses were harvested. Error bars represent standard deviation of the value obtained from triplicate culture bottles.

The growth and maximum biomass attained by *Symbiodinium* were influenced by both light intensity and spectral quality in two different Fe′ treatments (Fig 4). Comparison of growth curves for low light intensity treatments show that *Symbiodinium* subjected to red light have lower biomass compared to blue or white light cultures for most of the early stages until day 15, which coincided with the exponential growth phase (Fig 4A and 4B). When grown at low intensity of blue and white light, *Symbiodinium* reached about 3 × 10^5^ cells mL^-1^ after about 18 and 13 days of incubation in low and high Fe availability, respectively. For cultures grown using red light, biomass in low Fe treatment only reached about 1 × 10^4^ cells mL^-1^ compared to about 1 × 10^5^ cells mL^-1^ attained in high Fe treatment by the time that cultures in blue and white light treatments already reached the stationary phase. Comparing growth curves obtained in high light intensity, *Symbiodinium* grown in high intensity of blue and white light also reached about 3 × 10^5^ cells mL^-1^ after about 15 and 13 days of incubation in low and high Fe availability, respectively; while those grown in red light achieved biomass values reaching 2 × 10^5^ cells mL^-1^ after further 3–5 days (Fig 4C and 4D).

**Fig 5:**
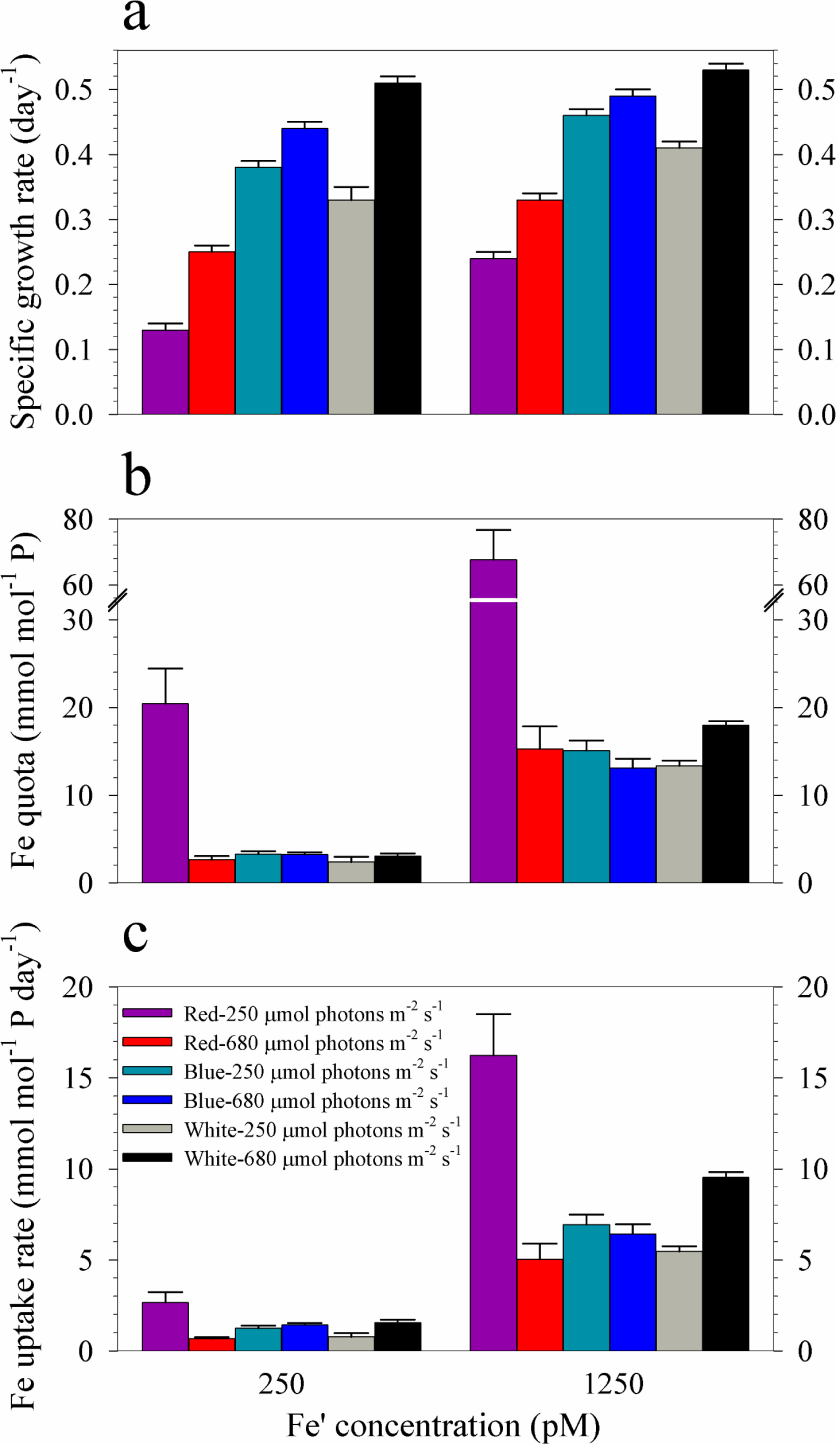
Specific growth rates, intracellular Fe quotas, and uptake rates of *Symbiodinium* cultures grown in blue, red or white light. **(a)** Specific growth rates, **(b)** Fe quotas, and **(c)** Fe uptake rates of cultures grown in light supplied at 250 or 680 μmol photons m^-2^ s^-1^ and 250 pM or 1250 pM Fe′. Error bars represent standard deviation of the value obtained from triplicate culture bottles.

**Fig 6:**
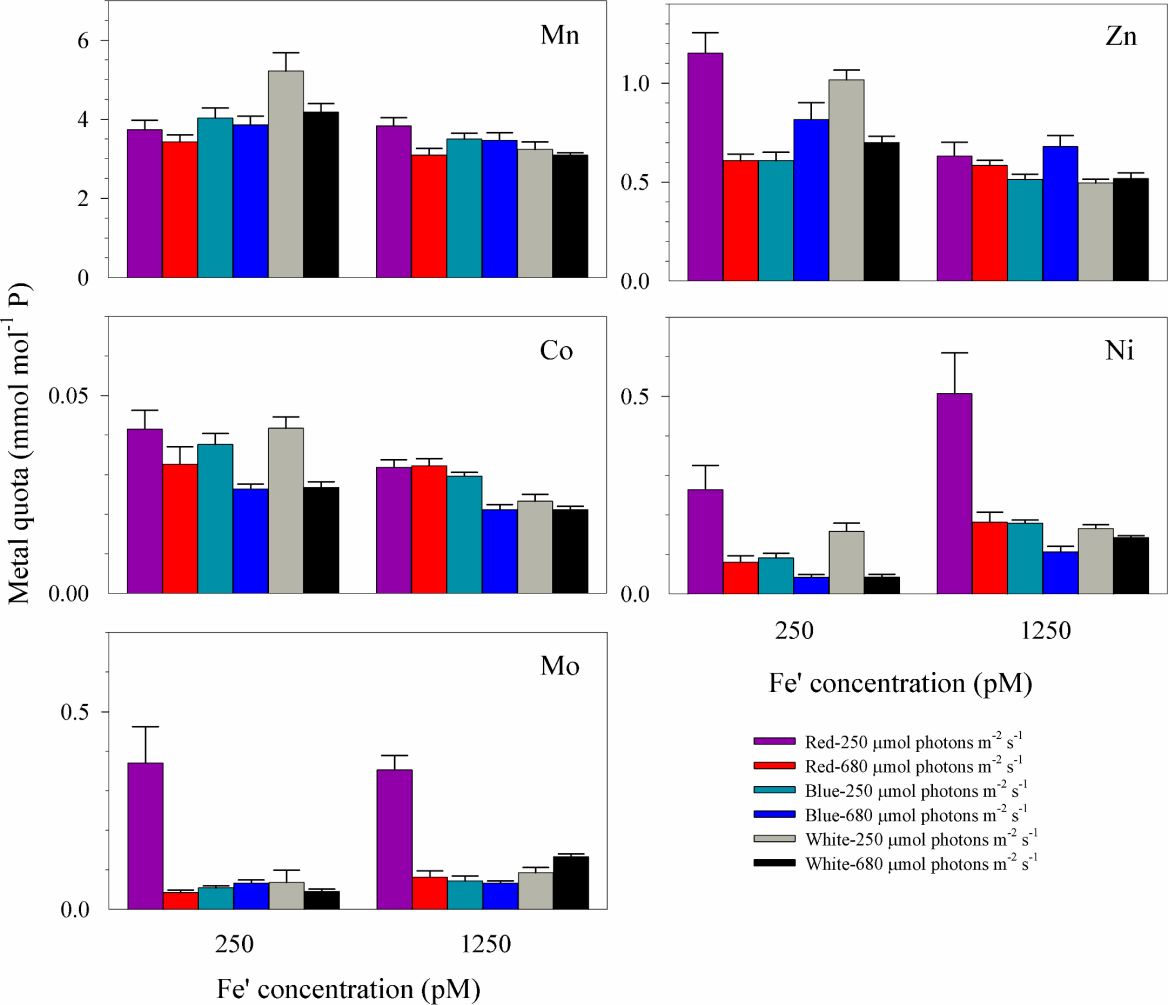
Trace metal quotas of *Symbiodinium* cultures grown in blue, red or white light. Cultures were subjected to light at 250 or 680 μmol photons m^-2^ s^-1^ and supplied with 250 pM or 1250 pM Fe′. Error bars represent standard deviation of the value obtained from triplicate culture bottles.

The growth rates were higher for high Fe′ treatments than in their corresponding low Fe′ treatments, except for cultures grown in high intensity white light where rates were not significantly different between high and low Fe treatments (Fig 5A, Table 2). In both sets of Fe treatments, rates increased congruently with light intensity for all spectral treatments. It is worth noting that in low light treatments, growth rates increased in the following order: red<white<blue light in both sets of Fe treatments. Whereas, the order observed in high light treatments was red<blue<white light in both sets of Fe treatments. Fe quotas reveal that intracellular Fe was highest in *Symbiodinium* supplied with low intensity red light among treatments supplied with equivalent Fe (Fig 5B, Table 2). Fe uptake rates show a trend similar to that observed in Fe quotas within different Fe treatments (Fig 5C, Table 2). In both the low and high Fe treatments, low red cultures had the lowest Fe-NUE with values of 0.0064 ± 0.0013 and 0.0035 ± 0.0005 mol P pmol^-1^ Fe day^-1^, respectively (Table 2). Among treatments with similar Fe availability and light intensity, red light cultures consistently showed the lowest Fe-NUE values. Intracellular metal quotas of other metals show that Mn and Co quotas in all treatments do not show a specific pattern with respect to either light quality or intensity (Fig 6). The Ni and Mo quotas, however, follow a pattern similar to that observed for Fe quotas. Both Ni and Mo quotas were elevated in cultures subjected to low intensity red light. Zn quota in low Fe-low red light cultures was also elevated but the value was comparable to others in the set with high Fe availability.

**Table 2:** Specific growth rates, intracellular Fe quotas, Fe uptake rates, and Fe net use efficiency of *Symbiodinium* cultures grown in blue, red or white light. The values represent means ± SD (*N* = 3, *P* < 0.05, ANOVA, post hoc LSD).

Treatment Number	Treatment description	Fe availability (Fe′, pM)	Light intensity (μE m^-2^ s^-1^)	Light quality	Growth rate (day^-1^)	Fe quota (mmol Fe mol^-1^ P)	Fe uptake rate (mmol Fe mol^-1^ P day^-1^)	Net Use Efficiency (mol P pmol^-1^ Fe day^-1^)
1	low Fe-low red	250	250	red	0.13 ± 0.01^c^	20 ± 4^b^	2.7 ± 0.6^b^	0.0064 ± 0.0013^b^
2	low Fe-low blue	250	250	blue	0.38 ± 0.01^b^	3.3 ± 0.3^a^	1.2 ± 0.1^a^	0.12 ± 0.01^a^
3	low Fe-low white	250	250	white	0.33 ± 0.02^a^	2.4 ± 0.6^a^	0.78 ± 0.20^a^	0.14 ± 0.03^a^
4	low Fe-high red	250	680	red	0.25 ± 0.01^c^	2.6 ± 0.4^a^	0.66 ± 0.10^a^	0.094 ± 0.015^c^
5	low Fe-high blue	250	680	blue	0.44 ± 0.01^b^	3.2 ± 0.2^a^	1.4 ± 0.1^a^	0.14 ± 0.01^a^
6	low Fe-high white	250	680	white	0.51 ± 0.01^a^	3.0 ± 0.3^a^	1.6 ± 0.2^a^	0.17 ± 0.02^a^
7	high Fe-low red	1250	250	red	0.24 ± 0.01^c^	69 ± 9^b^	16 ± 2^c^	0.0035 ± 0.0005^b^
8	high Fe-low blue	1250	250	blue	0.46 ± 0.01^b^	15 ± 1^a^	6.9 ± 0.6^b^	0.030 ± 0.002^a^
9	high Fe-low white	1250	250	white	0.41 ± 0.01^a^	13 ± 1^a^	5.5 ± 0.3^a^	0.031 ± 0.002^a^
10	high Fe-high red	1250	680	red	0.33 ± 0.01^c^	15 ± 3^a^	5.0 ± 0.9^c^	0.022 ± 0.004^a^
11	high Fe-high blue	1250	680	blue	0.49 ± 0.01^b^	13 ± 1^a^	6.4 ± 0.5^b^	0.037 ± 0.003^a^
12	high Fe-high white	1250	680	white	0.53 ± 0.01^a^	18 ± 1^a^	9.5 ± 0.3^a^	0.029 ± 0.001^a^

## Discussion

### *Trichodesmium* growth under varying light conditions and Ni′

Our study shows that light condition and Ni availability interact to influence the growth of *Trichodesmium* (Figs 1 and 2, and Table 1). When Ni supply was sufficient (Ni′ = 67 pM), *Trichodesmium* reached maximum growth rates in both high and low intensities of red, blue and white light. When Ni′ was limited (Ni′ = 6.7 pM) and light intensity was relatively low (250 μmol photons m^-2^ s^-1^), the growth followed the order: red<blue<white. This order of preference for light quality was in agreement with what was observed in a previous study on *Trichodesmium* grown using 40 μmol photons m^-2^ s^-1^ [13]. The substantial reduction of cyanobacterial growth in red light has also been reported by other researchers and was attributed to the effect of elevated ROS production and oxidative stress due to absorption of red light [29–31]. Hsieh and colleagues have observed excessive ROS production within cyanobacterial cells subjected to red light at intensities ranging from 50 to 100 μmol photons m^-2^ s^-1^ [29]. They found that red light supplied at 50 μmol photons m^-2^ s^-1^ resulted in 65–94% reduction in photosystem II quantum yields in seven cyanobacterial species while blue light with similar intensity hardly caused photoinhibition [29]. Our results coupled with the observation of Hsieh et al. support our previous hypothesis that Ni-SOD is involved in alleviating oxidative stress during photosynthesis in *Trichodesmium* [16,29]. In addition to SOD, there are two other Ni-containing enzymes in *Trichodesmium*, urease and NiFe-uptake hydrogenases [32,33]. The use of Ni in urease may be discounted since our culture media were free of urea. The activity of both Ni-SOD and NiFe-uptake hydrogenase may be pertinent in regulating cellular ROS levels enabling *Trichodesmium* to fix nitrogen. Further studies focusing on the connection between light conditions and expression of enzymes such as Ni-SOD and NiFe-uptake hydrogenase will provide an insight on the biochemical role of Ni in *Trichodesmium*.

We have also observed that the low Ni-low red and high Ni-high blue light treatments exhibit remarkable Ni quotas (Fig 2B, Table 1). Despite having the lowest growth rate among low Ni treatments, low red light cultures presented highest cellular quota, suggesting high Ni requirement or uptake. Among high Ni treatments, the high blue light treatment exhibited the highest growth rate but lowest Ni quota, suggesting that cells grown in this condition had low Ni requirement or uptake. Other than biochemical requirement, the variation in quotas in these treatments may be partially explained by the effect of growth dilution as a result of growth performance of *Trichodesmium* [34]. Specifically, the slow growth of low Ni-low red cultures resulted in minimum growth dilution of Ni and led to higher intracellular Ni content. Similarly, the fast growth rate observed in high Ni-high blue cultures may have resulted in lower intracellular Ni content through growth dilution. However, we did not observe systematic variation in metal quotas with growth rates among treatments, suggesting that the dilution effect is minor. The overall variations in uptake rates indicate that the biochemical Ni requirement is the primary mechanisms responsible for intracellular content (Fig 2C, Table 1). The evaluated Ni-NUE values further highlight the difference between low red cultures, which had the lowest value, with other low Ni treatments. Similarly, the high blue cultures had the highest Ni-NUE among high Ni treatments. The data for Ni quotas show that Ni uptake in *Trichodesmium* is highly dynamic and influenced by both light quality and intensity.

In our previous studies, we found that elevated Ni supply was essential for *Trichodesmium* to reach high growth rates and maximum biomass under high intensity white light [17,24]. Here, we also observed that the variations in nitrogen fixation activities in high Ni treatments were associated with intracellular Ni quotas and uptake rates in respective treatments (Figs 2 and 3, and Table 1). With high Ni availability, *Trichodesmium* fixed high amounts of nitrogen except the treatment subjected to high blue light. These results support our previous observations that elevated intracellular Ni concentrations are associated with enhanced nitrogen fixation activity in *Trichodesmium* [16,24]. It is worth noting that the high blue light treatment had the highest growth rate and Ni-NUE among cultures subjected to high Ni availability yet presented the lowest nitrogen fixation rates. We have validated the observation by repeating the experiment on this specific treatment. When Ni-NUE was calculated by considering the nitrogen fixations rates and Ni quotas (Ni-NUE-N_2_), the high blue cultures also had low Ni-NUE-N_2_ values. These results indicate that *Trichodesmium* may have mechanisms in place to favor biomass formation even in limited pool of fixed nitrogen. We propose that *Trichodesmium* under high blue light condition requires less nitrogen so that it only fixes the nitrogen required for its growth. Further study should include cellular nitrogen and carbon composition to validate whether *Trichodesmium* may sustain high growth with relatively low nitrogen fixation activities. For treatments at low light intensity, blue light cultures fixed higher amounts of nitrogen compared to both the white and red light cultures, a finding that is consistent with observations in a previous study [12]. The blue light cultures also had higher Ni-NUE-N_2_ compared to the other two treatments. At high light intensity, red light cultures fixed more nitrogen and had higher Ni-NUE-N_2_ compared to both the white and blue light cultures. These trends demonstrate that light quality plays an important role in nitrogen fixation by *Trichodesmium* and implies that it can utilize differing intensities of red and blue light, *i*.*e*., intense red light in surface waters and low intensity blue light that predominates in deeper waters.

The versatility to acclimatize to various light conditions provides *Trichodesmium* an advantage to survive in different depths. The adaptability of *Trichodesmium* is largely due to the flexibility in chromophore arrangement involving interconversion between phycourobilin and phycoerythrobilin upon exposure to differing light intensities in the water column [10,35]. Although *Trichodesmium* mostly exists in the surface water, its occurrence has also been observed in the nutricline, which can be as deep as 200 m [13,36]. Indeed, *Trichodesmium* has been reported to undergo vertical migration by controlling its buoyancy [37]. Since the availability of major and minor nutrients increases with depth, particularly below the nutricline, it is also likely that *Trichodesmium* sinks to deeper water to take up major and minor nutrients, such as P, Fe, and Ni. Typically, Ni concentration in the surface waters of the tropical and subtropical oceans is at 2 nM and increases with depth following a nutrient-type distribution. It is uncertain whether the 2 nM Ni is sufficient to promote maximum growth of *Trichodesmium* because the bioavailability of Ni in the surface water to the diazotroph is unknown [16]. Our results suggest that nutrient availability together with physical properties of light, *e*.*g*. spectral quality and intensity, may be a driving force influencing the spatial distribution of *Trichodesmium*. The buoyancy of *Trichodesmium* coupled with its ability to carry out nitrogen fixation and photosynthesis under high light condition bestow unique capabilities for it to sustain growth and become a dominant phytoplankton group in the oligotrophic tropical and subtropical oceans.

### *Symbiodinium* growth under varying light conditions and Fe′

Our previous study revealed that cellular Fe demand is extremely high in *Symbiodinium*, requiring 500 pM Fe′ for *S*. *kawagutii* to reach maximum growth rate when all other essential trace metals are sufficient [24]. In this study, we have also provided sufficient Zn, Cu, Mn, Co, and Ni in culture media but varied Fe′ to investigate the interactive effect of light condition and Fe′ on *Symbiodinium* growth. In terms of measurement of Fe quotas, it should be noted that extracellularly adsorbed Fe may possibly contribute to total Fe quotas obtained for high Feʹ treatments since filtered cells were only rinsed with ultrapure water. However, several lines of evidence show that the contribution of extracellular Fe should be minor in cells grown under high Feʹ culture medium. First, the metal quotas observed in high Feʹ treatments, ranging from 13 to 18 mmol mol P^-1^ (Table 2), were comparable to intracellular Fe quotas observed in systematic culture studies conducted under similar Feʹ wherein extracellular wash was done on cells to remove adsorbed or precipitated Fe [38,39]. Second, Ni and Mo quotas varied in a pattern similar to that observed for Fe among treatments with high and low Feʹ availability indicating that the variations of the three metal quotas were likely due to biochemical requirement (Figs 5 and 6). Third, similar levels of elevated Fe quotas were also observed in *Symbiodinium* grown under extremely low Cuʹ and Znʹ conditions indicating replacement strategy or functional complementarity employed by the dinoflagellate [24]. All of these observations suggest that Fe quotas of *Symbiodinium* measured in this study were mainly intracellular and may thus be attributed to biological requirement.

Consistent with our previous finding [24], treatments with high Feʹ (1250 pM) resulted in significantly higher growth rates compared to corresponding treatments with low Feʹ (250 pM; Fig 5A and Table 2). In terms of light quality, red light cultures have lower growth rates compared to blue and white light treatments with similar light intensity and Fe availability. The lowest growth rates were observed in low red light cultures in both low and high Fe′ treatments with values of 0.13 ± 0.01 and 0.24 ± 0.01 day^-1^, respectively. These results are consistent with previous observations that red light induced slower growth of dinoflagellates compared to blue light [14,15]. However, unlike the effect of Niʹ on *Trichodesmium*, elevated Fe′ does not fully relieve the growth stress of *Symbiodinium* under red light conditions. We propose that the different responses between *Trichodesmium* and *Symbiodinium* under red light may be attributed to the presence of red light absorption pigments in *Trichodesmium*. Similar to other cyanobacteria, *Trichodesmium* possesses phycocyanin and phycoerythrin, which are unique pigments that confer cyanobacteria the advantage to absorb much wider range of red and green light.

In this work, we observed a positive correlation with increase in light intensity on the dinoflagellate growth for all spectral treatments in both low and high Fe treatments (Fig 5A and Table 2). We have also observed that growth rates were higher under high Feʹ treatments than in low Feʹ treatments under same light conditions. Shallow water coral reef ecosystems experience dramatic light fluctuations with intensities generally higher than 2000 μmol photons m^-2^ s^-1^ during noon time in the tropical and subtropical regions. Although intracellular downwelling light in coral may be reduced by one order of magnitude [40], symbiotic dinoflagellates in corals may receive light with much higher intensity than the downwelling light because incident light reaching the coral endoderm may be scattered and amplified by coral skeleton microstructure [41,42]. The positive influence of higher light intensity and Feʹ on the dinoflagellate growth observed in this study indicates that sufficient Fe bioavailability is critical for *Symbiodinium* to survive in high light environment.

The Fe quotas observed in blue and white light treatments were comparable under same Feʹ availability ranging from 2.3 to 3.2 or 13 to 18 mmol mol P^-1^ when Feʹ was 250 or 1250 pM, respectively (Fig 5B, Table 2). The Fe quotas in high Feʹ treatments were either comparable to or slightly lower than values observed previously in treatments with Feʹ amended at 2500 pM [24]. In contrast to quotas observed in blue and while light treatments, we found that Fe quotas observed in low red light treatments were remarkably high, increasing by a factor of 4 in comparison to values in cultures with equivalent Fe′. Fe uptake may be influenced by changes in Fe bioavailability due to photochemical dissociation of Fe-EDTA complexes [43]. In a previous work by Sunda and Huntsman (1995), they found that 20–25% of Fe′ increases when light intensity increased from 175 to 500 μmol photons m^-2^ s^-1^ [39]. This suggests that the contribution of photoreduction to our quotas is limited as demonstrated by the comparable Fe quotas between high- and low-light treatments under same Fe′ condition. We also observed strikingly high Fe quotas in low red light cultures, which were not observed in high red light counterparts, in both sets of Fe treatments. The calculated Fe-NUE values in low red light cultures were also the lowest in both sets of Fe treatments. Interestingly, red light cultures had lower Fe-NUE values compared to blue and white light cultures subjected to similar Fe and light intensity. These results indicate that Fe uptake may be influenced by coupled effect of intensity and quality of light. The high Fe quotas in low red light cultures may have resulted from increased requirement or partially from lowered growth dilution of Fe because of slow growth of *Symbiodinium* in these treatments [34]. It should be noted that red light can be a dominant or major light source for *Symbiodinium* in corals [40]. The elevation of intracellular Fe under low red light condition implies that Fe availability is an important factor influencing the growth of *Symbiodinium* under low red light condition. The mechanisms causing elevated quotas for the low red light treatments certainly warrant further studies.

Compared to Fe quotas shown in Fig 5B, the intracellular quotas of Mn and Co were comparable among all twelve treatments with varying light qualities and Fe′ concentrations (Fig 6), with averaged Mn and Co quotas of 3.7 ± 0.6 and 0.031 ± 0.007 mmol mol^-1^ P, respectively. The comparable quotas indicate that the requirement for Mn- and Co-containing metalloproteins were independent of light quality and Fe availability and that intracellular P was relatively stable. The overall averaged Zn and Ni quotas were 0.76 ± 0.23 and 0.078 ± 0.050 mmol mol^-1^ P, respectively. We have noticed that Zn and Ni quotas were elevated by about 2 to 3 folds in low Fe-low red light cultures in comparison with low Fe-high red light cultures. The elevated Zn and Ni quotas may be due to lower growth dilution of these metals owing to inhibited growth of *Symbiodinium* in low Fe-low red light condition [34]. However, the comparable Mn and Co quotas observed in all treatments suggest possibility of other mechanisms resulting in increased Zn and Ni quotas in low Fe-low red light cultures. The elevated quotas may be attributed to increased cellular requirement to counter low Fe availability although it is also likely due to overexpression of non-specific divalent metal membrane transporters [24]. Similar to Fe, Mo quota in low Fe-low red light treatment was elevated to 0.37 ± 0.09 mmol mol^-1^ P and the value in high Fe-low red light treatment was elevated to 0.35 ± 0.04 mmol mol^-1^ P. These values were about 5 folds of the averaged value, 0.07 ± 0.02 mmol mol^-1^ P, in all other treatments regardless of Fe availability or light quality and intensity. Mo enzymes in dinoflagellates include xanthine dehydrogenase, an enzyme involved in free radical metabolism in dinoflagellates [44]. Aside from xanthine dehydrogenase, Mo is also present in nitrate reductases [45]. The dinoflagellate was supplied with nitrate as its sole nitrogen source in this study and it is possible that Mo utilization was mainly involved in nitrogen assimilation. The observed co-variation in Mo and Fe quotas under low intensity of red light warrants further studies to identify whether Mo also contributes to anti-oxidative defenses of the cell.

## Conclusion

Marine photosynthetic organisms are subjected to variable light intensity and quality in combination with rapid changes of major chemical growth factors including the supply of essential trace metals. We hypothesize that the availability of specific trace metals may play important roles in regulating the growth of *Trichodesmium* and *Symbiodinium* under various light qualities. Our results show that light quality and quantity interact with trace metal availability to influence the growth of *Trichodesmium* and *Symbiodinium*. In controlled laboratory conditions, the exposure to low intensity red light increases Ni quota in *Trichodesmium* and increases Fe uptake in *Symbiodinium*. Nitrogen fixation by *Trichodesmium* was influenced by interactive effects of quality and quantity of light with reduction in nitrogen-fixing activity in high intensity blue light condition as the most pronounced observation. We found that high Ni availability relieves the growth inhibition of *Trichodesmium* under red light while high Fe availability only partially relieves similar stress in *Symbiodinium*. Our work demonstrates that availability of specific trace metals may play important yet different roles in regulating growth of *Trichodesmium* and *Symbiodinium* under various light qualities and particularly under low red light condition. The concerted effects of light intensity and quality compounded with trace metal availability may influence the growth of photosynthetic organisms in the ocean. For future studies, measurement of cellular ROS activity and SOD expression in the microalgae grown under various intensities of red light and trace metal supply may shed light on mechanisms pertaining to how light quality and metal availability interact to influence growth of important photosynthetic organisms.
